# Supplementary material for: Homozygous frameshift mutations in FAT1 cause a syndrome characterized by colobomatous-microphthalmia, ptosis, nephropathy and syndactyly
Source: Nat Commun. 2019 Mar 12;10:1180. doi: 10.1038/s41467-019-08547-w (PMC6414540; doi:10.1038/s41467-019-08547-w)
Supplement: Supplementary file 2 — Supplementary Information [file 41467_2019_8547_MOESM2_ESM.pdf]

Supplementary information file

Homozygous frameshift mutations in FAT1 cause a syndrome characterized by colobomatous-microphthalmia, ptosis, nephropathy and syndactyly

Lahrouchi, George and Ratbi et al.

Supplementary Figures 1-11

Supplementary Tables 1-2

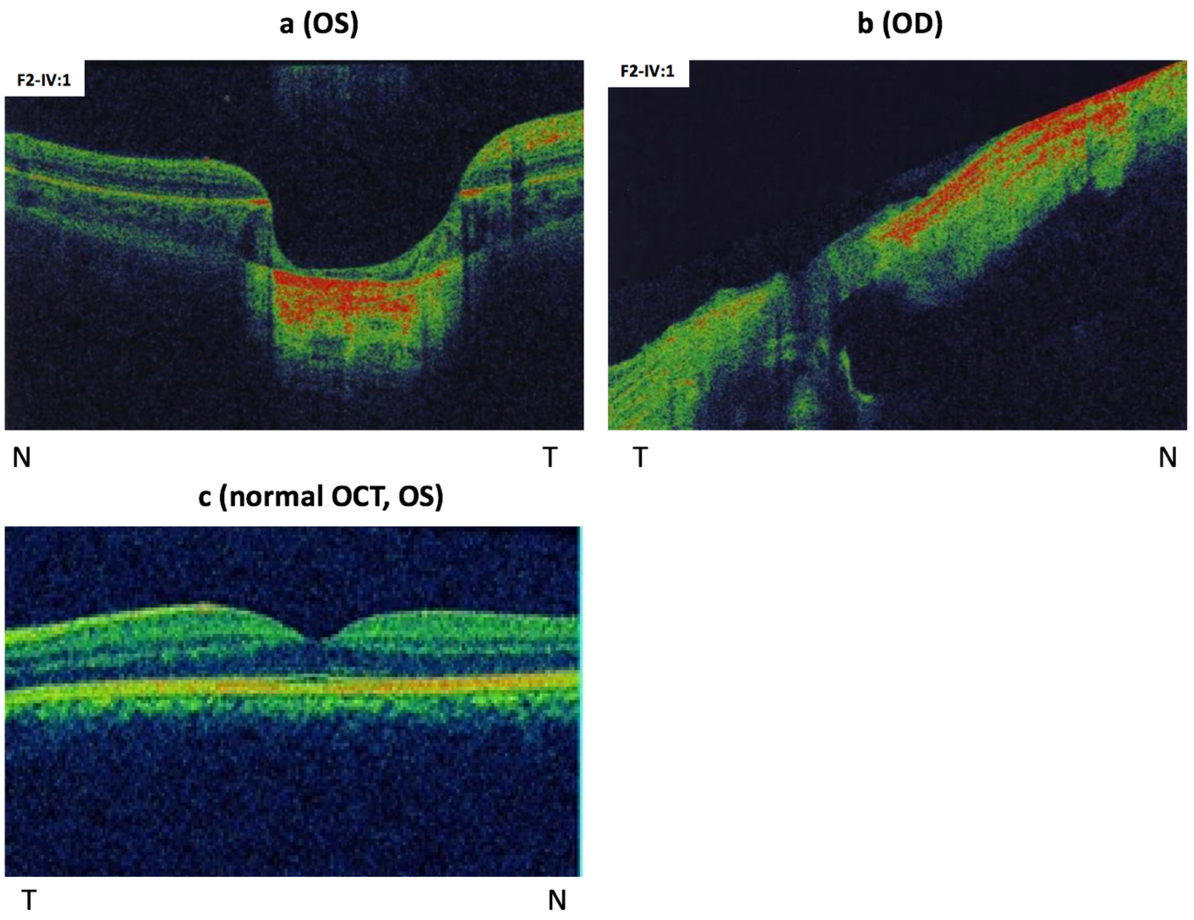

Supplementary Figure 1. Optical coherence tomography (OCT) of individual F2-IV-1. In the right eye (OD), a horizontal cross-section through the coloboma is shown with a large colobomatous fossa, extensive retinoschisis associated with retinal cysts and a serous retinal detachment were also seen in on other cuts (**a**). In the left eye (OS) a small colobomatous fossa without retinoschisis nor serous retinal detachment is seen (**b**). Normal OCT image for comparison (**c**). Abbreviations: T, temporal and N, nasal.

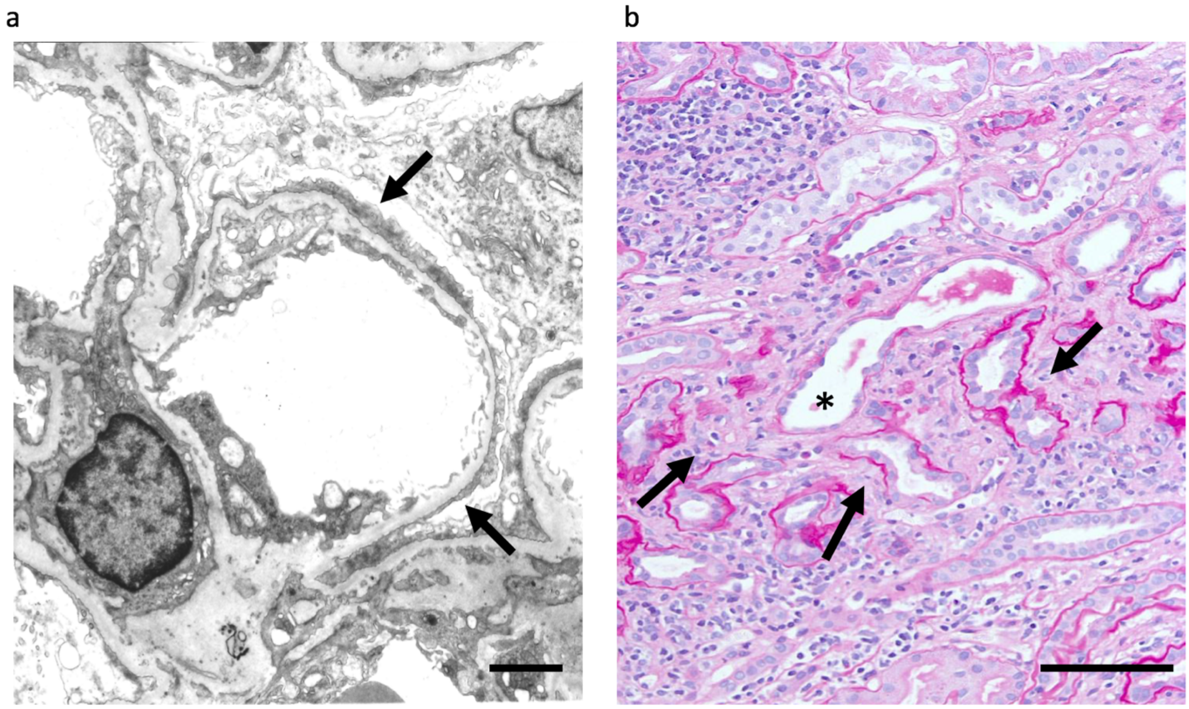

Supplementary Figure 2. Renal biopsy images of patient F5-II-1.

Electron microscopy in F5-II-1 demonstrates the common nephrotic syndrome (NS) feature of foot process effacement (**a,** arrows). Scale bar, 5 mm. Renal histology of patient F5-II-1 exhibits cystic dilation of renal tubules (hash), interstitial infiltrations (asterisks), and tubular basement membrane disruptions (**b**, arrowheads). Scale bar, 100 mm. Images have been previously published elsewhere.[8]

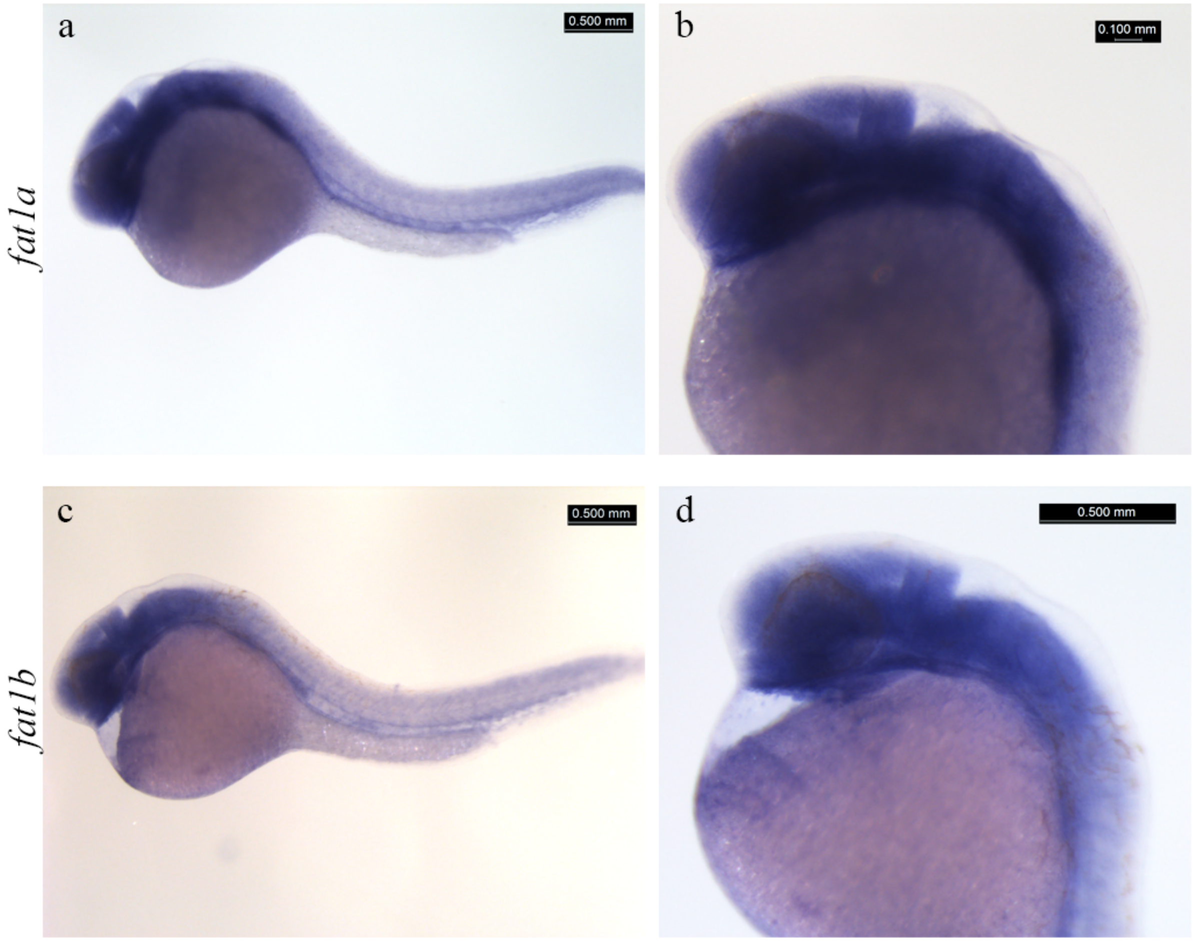

Supplementary Figure 3. *In situ* staining of zebrafish embryos.

In situ staining of zebrafish embryos (24 hpf) with *fat1a* (**a and b**) and *fat1b* (**c and d**) riboprobes. Scale bar is 0.5mm.

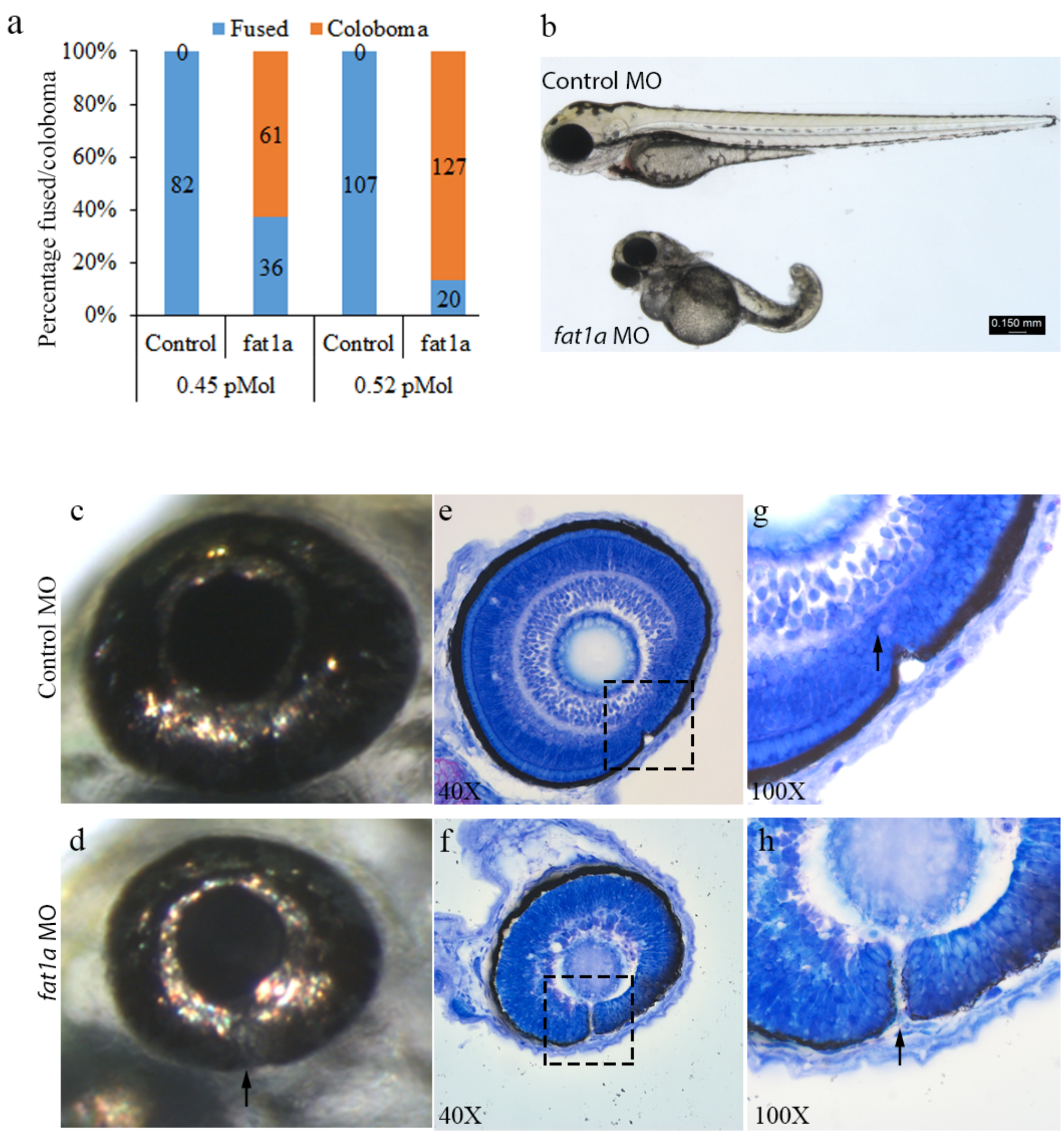

Supplementary Figure 4. Morpholino mediated knockdown of zebrafish *fat1a* causes coloboma

Morpholino mediated knockdown of *fat1a* consistently resulted in coloboma at two different concentrations (**a, b, d, f and h**), whereas injection of similar amount of control morpholino did not cause coloboma (**a, b, c, e and g**). Fused optic fissure margins of zebrafish larvae (day 3 post fertilization) can be observed (**e and g; sagittal section**) after histology and toludene blue staining, whereas the fissure margins remained unfused in *fat1a* morphant embryos (**f, and h**).

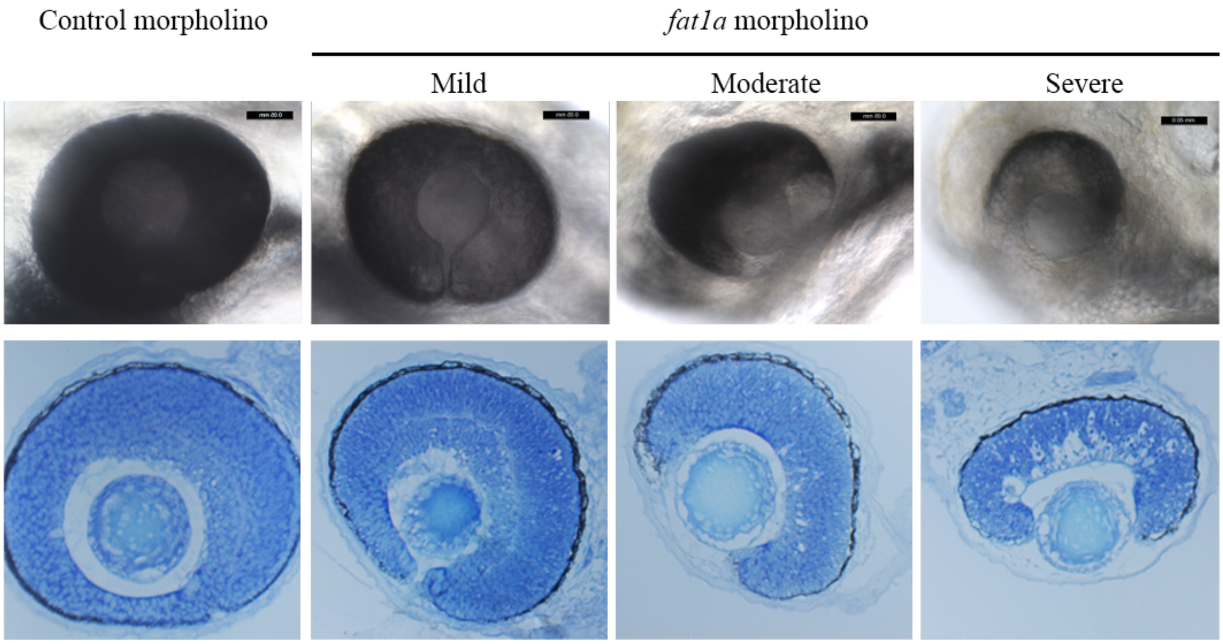

Supplementary Figure 5. Variability of coloboma phenotype in *fat1a* morphant zebrafish embryos.

Optic cup morphology of control and *fat1a* morphant zebrafish embryos. Top panel shows the variability in coloboma phenotype ranging from mild to severe followed by histology in the bottom panel.

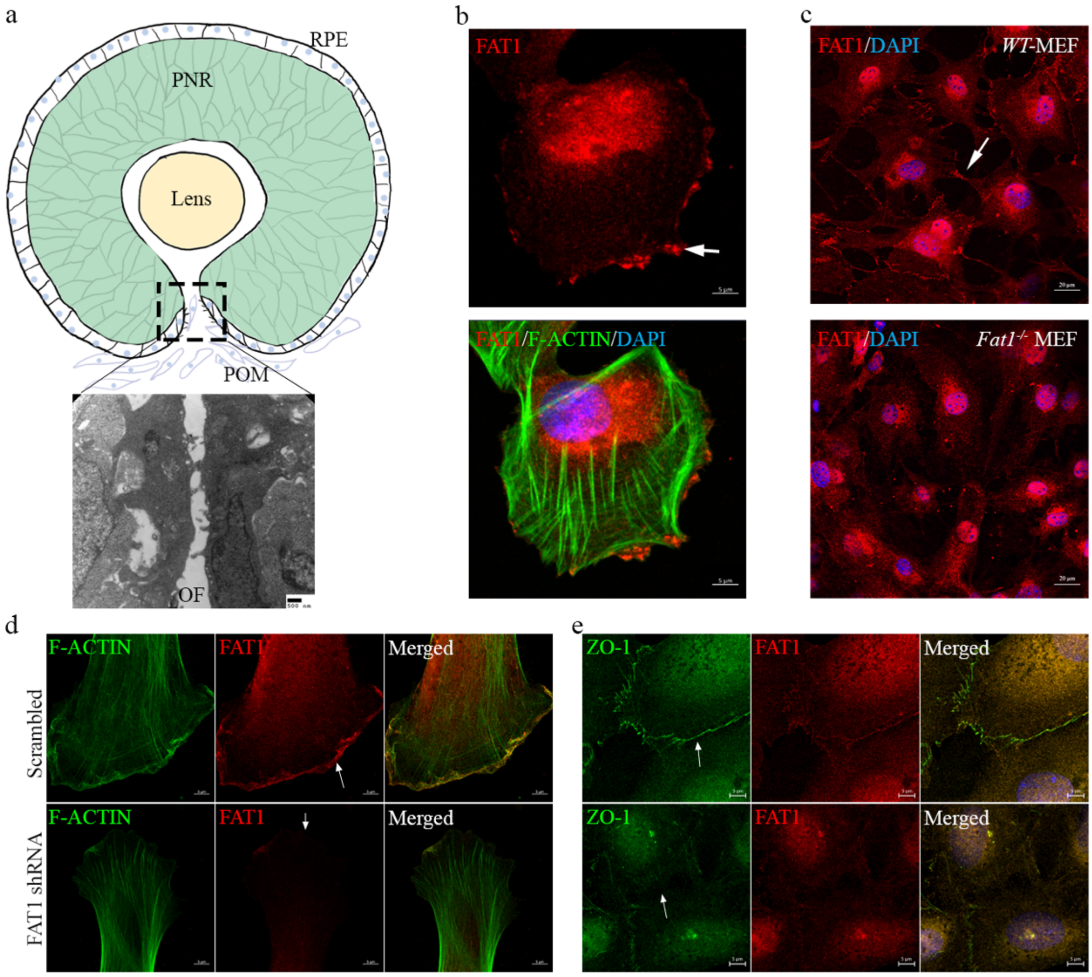

Supplementary Figure 6. FAT1 localization at leading edges of cell observed by immunostaining

Schematic representation of the optic cup during time of optic fissure closure (**a**, **top panel**). TEM micrograph showing cellular processes emanating from RPE at optic fissure margins before fusion in E11.5 mouse embryo (**a, bottom panel**). FAT1 immunostaining was observed at the leading edges of isolated RPE cells (**b, arrow, scale bar is 5**µm) and mouse embryonic fibroblasts isolated from WT and *Fat1*$^{-/-}$ mice (**c, arrow, scale bar is 20**µm). shRNA mediated knockdown of FAT1 resulted in loss of FAT1 from filopodia (**d, scale bar is 5**µm) and loss of ZO-1 from earliest cell-to-cell contacts (**e, scale bar is 5**µm). **Abbreviations**: RPE, retinal pigment epithelium; PNR, presumptive neural retina; POM, peri-ocular mesenchyme and OF, optic fissure.

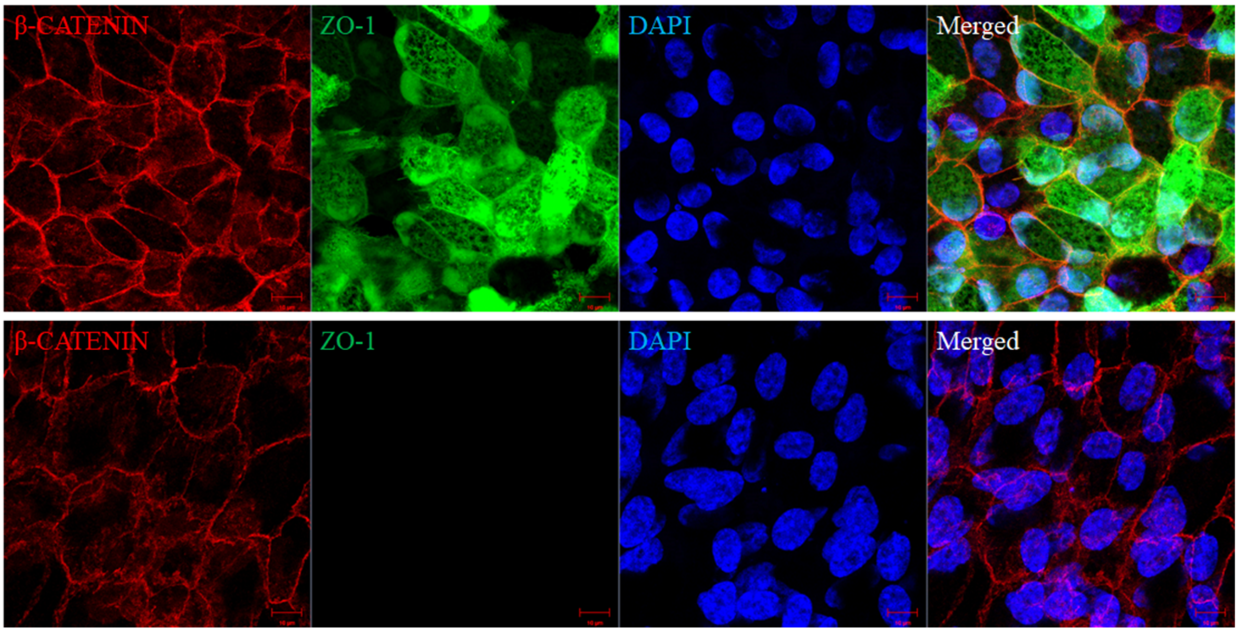

Supplementary Figure 7. Localization of β-Catenin and ZO-1 in RPE cells

Immuno-staining for β-Catenin and ZO-1 in RPE cells treated with scrambled (**top panel**) and FAT1 (**bottom panel**) shRNA. Scale bar is 20μm.

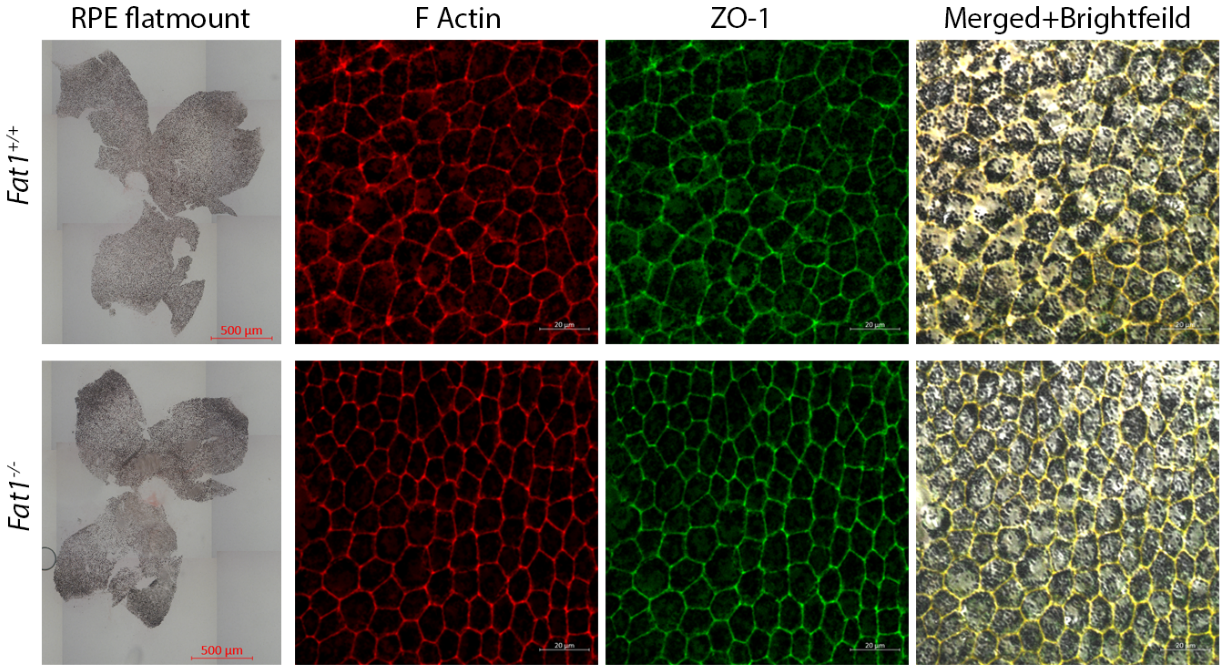

Supplementary Figure 8. Localization of F-Actin and ZO-1 in mouse RPE flat-mounts

Immuno-staining of RPE flat-mounts (scale bar is 500µm) prepared from WT (**top panel**) and Fat1$^{-/-}$ (**bottom panel**) mouse optic cups with F-Actin and ZO-1 (scale bar is 20µm).

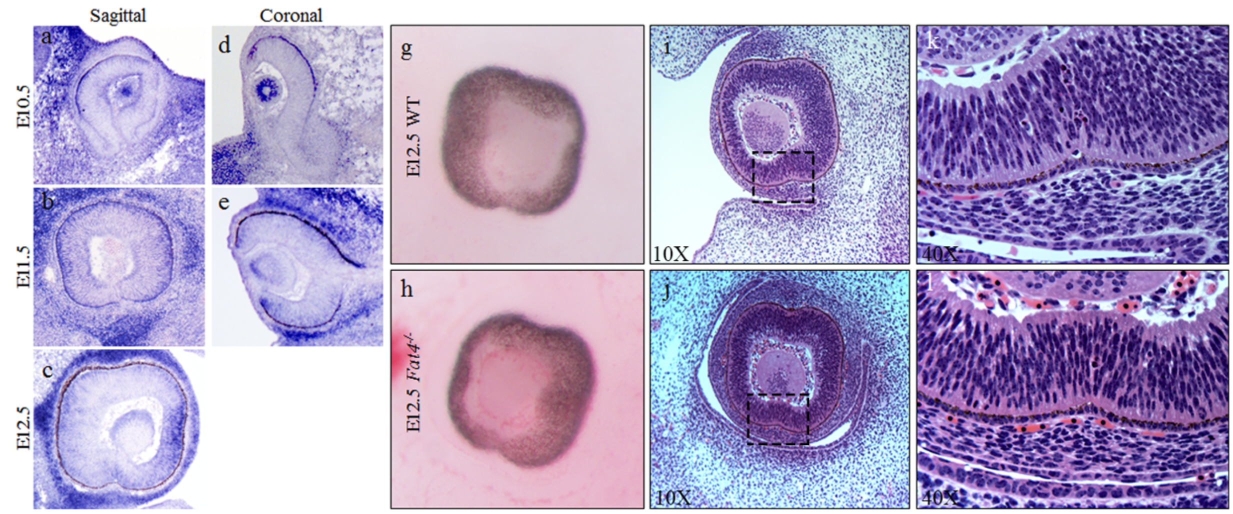

Supplementary Figure 9. Localization of *Fat4* mRNA in WT mouse optic cup and histology of WT and *FAT4*$^{-/-}$ mouse embryos

Fat4 mRNA expression was observed to be expressed at the apposing edges of fissure, in the lens, in the retina, and in the periocular mesenchyme (**a-e**). This expression was similar to the expression pattern of FAT1. Gross morphological and histological study performed on E12 WT (**g, i and k**) and *FAT4*$^{-/-}$ mouse (**h, j and l**) revealed no optic fissure closure defects in *FAT4*$^{-/-}$ mouse. Optic fissure margin in both WT and *FAT4*$^{-/-}$ mouse were fused by E12.5 (**k and l**).

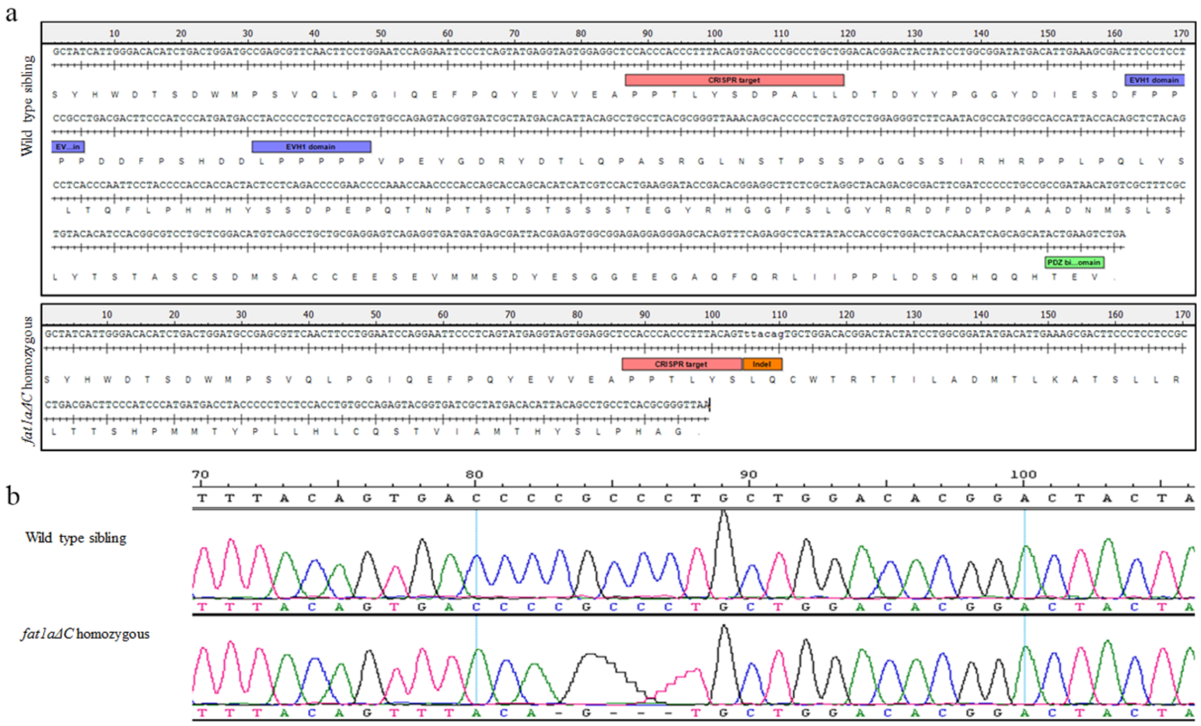

Supplementary Figure 10. Sequence chromatograms of CRISPR/Cas9 target in zebrafish *fat1a*

CRISPR/*Cas9* was used to generate frame shift mutations in the cytoplasmic domain of *fat1a* to disrupt the ENA/VASP and PDZ binding domains (**a**). We cannot exclude possibility of nonsense mediated decay (NMD), even though the mutant stop codon resides in the last exon thereby minimizing the chances of NMD. Sequence chromatograms showing frameshift deletion in zebrafish *fat1a* (**b**).

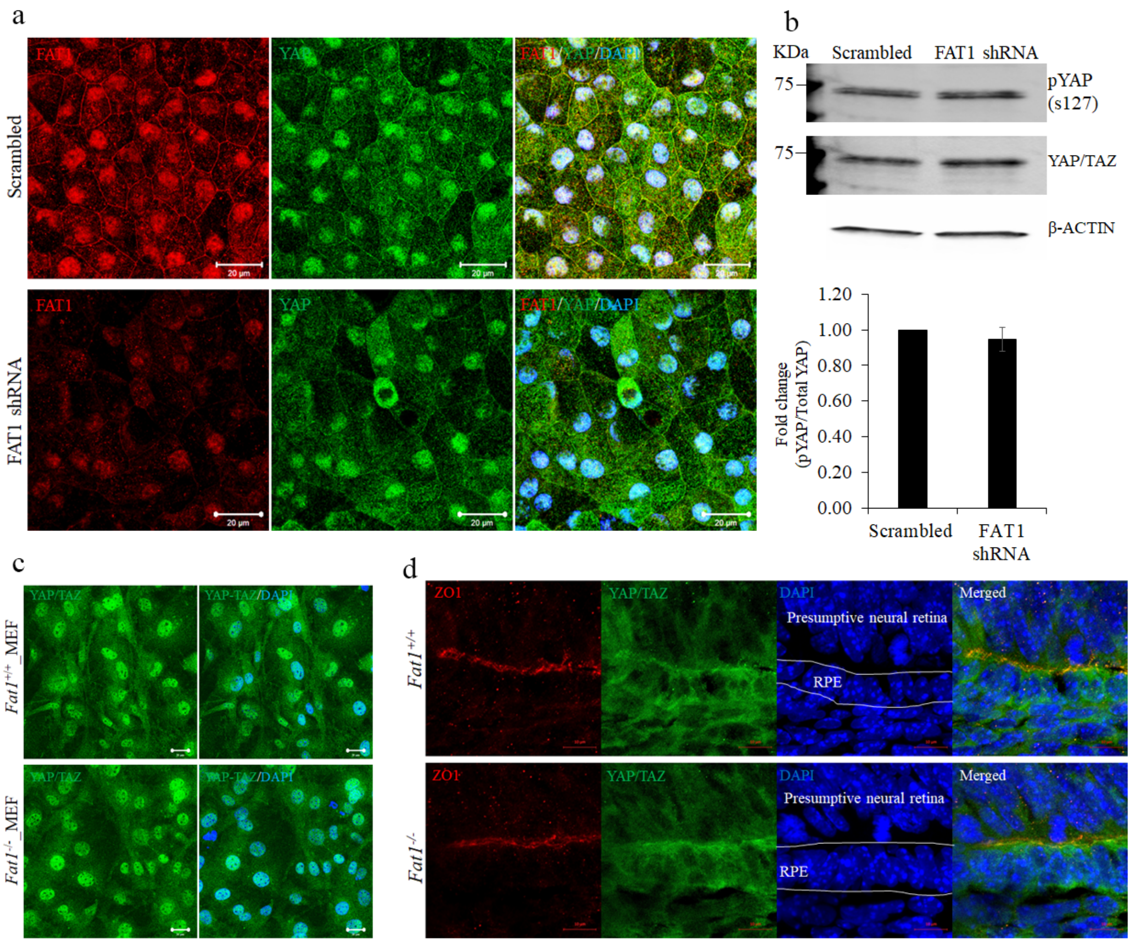

Supplementary Figure 11. Effect of FAT1 knock-down on YAP cellular localization and expression pattern.

FAT1 and YAP/TAZ immuno-staining of RPE monolayers (8 weeks post seeding) treated with scrambled and FAT1 shRNA for one week (**a,** scale bar is 20µm). Total YAP/TAZ, phosphorylated YAP (Ser127) and β-ACTIN protein levels in RPE cells treated with scrambled and FAT1 shRNA (**b, N=3**). Immuno-staining of mouse embryonic fibroblast cells with YAP/TAZ (**c,** scale bar is 20µm) and optic cup (E11.5-12.5) sections with ZO-1 and YAP/TAZ (**d,** scale bar is 10µm). Error bars represent standard error of mean. Raw data is provided in supplementary section.

Supplementary Table 1. Summary of sequencing protocols and mean depth of coverage for each family

	**Family 1**	**Family 2**	**Family 3**	**Family 4**	**Family 5**
**Exome Capture**	SureSelect Human All Exon kit V5 (Agilent technologies Inc)	SureSelect Human All Exon kit V5 (Agilent technologies Inc)	SureSelect Human All Exon kit V4 (Agilent technologies Inc)	NimbleGen SeqCap EZ Human Exome Library (Roche, NimbleGen Inc)	SureSelect Human All Exon kit V5 (Agilent technologies Inc)
**Sequencing**	HiSeq 2500 (Illumina)	HiSeq 2000 (Illumina)	HiSeq 2500 (Illumina)	HiSeq 2000 (Illumina)	HiSeq 2000 (Illumina)
**Mean coverage**	140X	50X	84X	90X	50X
**Consanguinity**	Yes	Yes	Yes	Yes	Yes
***FAT1* variant (all homozygous)**	c.2207dupT (p.I737NfsX7)	c.2207dupT (p.I737NfsX7)	c.2600_2601delCA (p.T867IfsX4)	c.9729del (p.V3245LfsX25)	c.3093_3096del (p.P1032CgsX11)

Supplementary Table 2. Full overview of clinical characteristics of homozygous FAT1 mutations carriers

	**Family 1**			**Family 2**			**Family 3**		**Family 4**	**Family 5**
	**IV:1**	**IV:3**	**IV:5**	**III:2**	**IV:1**	**IV:3**	**IV:1**	**IV:3**	**II:3**	**II:1**
***FAT1* mutation (All homozygous)**	c.2207dupT (p.I737NfsX7)			c.2207dupT (p.I737NfsX7)			c.2600_2601delCA (p.T867IfsX4)		c.9729del (p.V3245LfsX25)	c.3093_3096del (p.P1032CfsX11)
**Ethnicity**	Morocco			Morocco			Middle-East		Pakistan	Turkey
**Consanguinity**	+			+			+		+	+
**Sex**	F	M	M	F	F	M	M	M	M	M
**Age (years)**	39	34	18	36	8	2	27	24	8	8
**Facial dysmorphism**										
Long face	+	+	+	+	+	-	-	-	+	-
Highly arched eyebrows	+	+	+	+	+	+	-	-	+	-
Long nose	+	+	+	+	+	-	-	-	+	-
Low insertion of columella	+	+	+	+	-	-	-	-	-	-
Long philtrum	+	+	+	+	+	+	-	-	-	-

Thin upper lip	+	+	+	+	+	+	-	-	-	-
**Intellectual disability**	-	-	-	-	-	-	-	+	+	+
**Ocular features**										
Iris coloboma	-	-	B	U	-	B	-	-	-	-
Retinal coloboma	B	-	B	B	B	B	-	U	-	-
Ptosis	B	B	B	B	U	B	-	B	U	B
Strabism	-	-	U	U	-	U	-	-	U	-
Nystagmus	-	-	B	-	-	-	-	U	-	-
Photophobia	+	+	+	-	-	-	-	-	-	-
Microphtalmia	-	-	B	-	U	U	-	U	-	-
Cataract	-	-	U	U	-	-	-	-	-	-
Amblyopia	+	+	+	-	+	+	-	-	-	-
**Hands abnormalities**										
Clinodactyly	-	-	B	-	-	-	-	-	+	-
**Feet abnormalities**										

Syndactyly	-	3rd-4th RF, 3rd-4th LF	3rd-4th RF, 3rd-5th LF	3rd-4th RF	2nd-3rd RF	1st-2nd LF, 3rd-4th RF	3rd-4th RF; Bone fusion	3rd-4th RF; Bone fusion; phalanx hypotrophy	4th-5th bilaterally	-
Club feet	-	-	-	-	B	-	n/a	n/a	-	
**Renal manifestations**										
Nephropathy	+	-	+	-	-	-	+	+	-	+
Biopsy (at age)	n/a	n/a	n/a	n/a	n/a	n/a	FSGS (20 years of age)	n/a	n/a	TIN, MS, thin GBM (12 years)
**Others**	-	-	-	-	-	-	Renal artery stenosis	-	Immunological abnormalities, profound T-cell defects and recurrent infections	Pulmonary stenosis, pachygyria

Abbreviations are as follows: B, bilateral; F, female; FSGS, focal segmental glomerulosclerosis; GBM, glomerular basement membrane; LF, left foot; M, male; MS, mesangial sclerosis; n/a, not assessed; U, unilateral; RF, right foot, TIN, tubular interstitial nephritis; +, phenotype present; -, phenotype absent.
